# Central retinal artery occlusion following COVID-19 vaccine administration

**DOI:** 10.1016/j.ajoc.2022.101430

**Published:** 2022-02-18

**Authors:** Alaa Din Abdin, Barbara C. Gärtner, Berthold Seitz

**Affiliations:** a Department of Ophthalmology, Saarland University Medical Center UKS, Homburg/Saar, Germany; b Department of Medical Microbiology and Hygiene, Saarland University Medical Center UKS, Homburg/Saar, Germany

## Abstract

**Introduction:**

Increased risk of thromboembolic events has been associated with SARS-CoV-2 infections and more recently, with COVID-19 vaccination. To date, however, there are no reports of an association between the COVID-19 vaccination and retinal artery occlusions. We report a case of a patient who developed central retinal artery occlusion (CRAO) 2 days following the administration of the AstraZeneca COVID-19 vaccine.

**Case description:**

A 76-year-old woman presented to our Department of Ophthalmology complaining of painless vision loss in her left eye 48 hours after she had received her first dose of the AstraZeneca COVID-19 vaccine. Her best-corrected visual acuity was only hand movement in the left eye. Left eye ophthalmologic examination showed the presence of arterial narrowing and a cherry red spot. Optical coherence tomography showed severe macular swelling of the inner retinal layers in the left eye Fluorescein angiography performed the following day confirmed the diagnosis. The cardiovascular examination including Holter ECG was unremarkable. Complete blood count was within normal limits, without thrombocytopenia. A subsequent cerebral CT and CT-angiography scans did not show any other acute vascular event. Doppler angiography of the carotid artery was performed and showed normal flow without clinically significant plaques, stenoses, occlusions or dissections.

**Conclusions:**

To our knowledge, this is the first case of an isolated CRAO following the administration of the AstraZeneca COVID-19 vaccine. Further studies are needed to evaluate this potential association and identify pathophysiologic relationships between COVID-19 vaccinations and CRAO.

## 1 Introduction

Increased risk of thromboembolic events has been associated with SARS-CoV-2 infections^1^ and more recently, with COVID-19 vaccination.^2^ In particular, several reports have studied the association between cerebral venous thrombosis and the vector-based AstraZeneca COVID-19 vaccine (Vaxzevria) administration.^2^ To date, however, there are no reports of an association between the COVID-19 vaccination and retinal artery occlusions (RAO), which have unique characteristics and pathogenesis.^3^ We report a case of a patient who developed central retinal artery occlusion (CRAO) 2 days following the administration of the AstraZeneca COVID-19 vaccine.

## 2 Case Description

A 76-year-old woman presented to our Department of Ophthalmology complaining of painless vision loss in her left eye 48 hours after she had received her first dose of the AstraZeneca COVID-19 vaccine. The patient was known to have hypothyroidism, but no previous history of cardiovascular disease, hypercoagulable state, malignancy or diabetes.

**Fig. 1:**
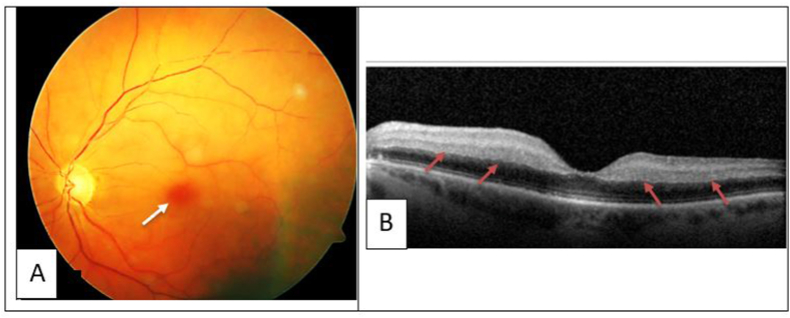
**Clinical and optical coherence tomography findings: (A)** Dilated fundus ophthalmoscopy showed the presence of arterial narrowing with cherry red spot (white arrow). (**B)** Optical coherence tomography showed severe macular swelling of the inner retina layers (red arrows). (For interpretation of the references to colour in this figure legend, the reader is referred to the Web version of this article.)

**Fig. 2:**
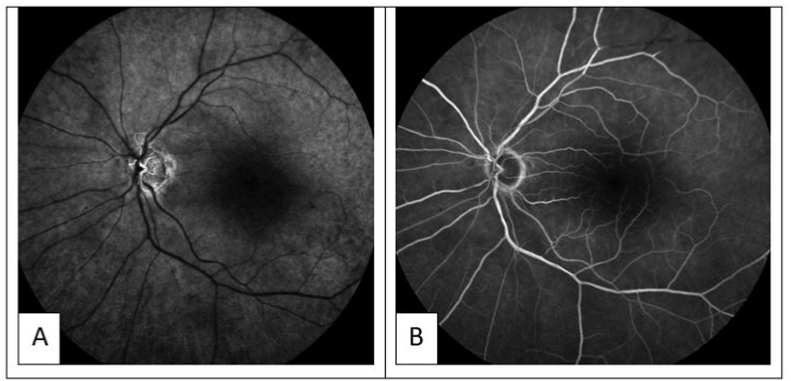
**Fluorescein angiography findings:** It showed **(A)** severe delay in the filling of the retinal arteries in the early arterial phase (16 seconds) and **(B)** central obscuring of the background choroidal fluorescence by retinal swelling in the late phase (5 minutes).

A nasopharyngeal swab (PCR test) on admission ruled out an ongoing COVID-19 infection. Her best-corrected visual acuity was 20/20 in the right eye, but only hand movement in the left eye. Left eye ophthalmologic examination showed a nonreactive mydriasis, and dilated fundus ophthalmoscopy revealed the presence of arterial narrowing and a cherry red spot with central retinal whitening. Optical coherence tomography showed severe macular swelling of the inner retinal layers in the left eye (Fig. 1 A, B). Fluorescein angiography performed the following day confirmed the diagnosis of CRAO evident by a severe delay in the filling of the retinal arteries in the early arterial phase and an obscuring of the background choroidal fluorescence by retinal swelling in the late phase (Fig. 2 A, B).

The patient was, therefore, admitted for inpatient management and was treated with:

• Ocular massage
• 500 ml Pentoxifylline i.v. once/day
• Dorzolamide eye drop 3 times/day as a hypotensive agent.
• Aspirine 100 mg once/day

The cardiovascular examination including normal ECG and Holter ECG was unremarkable. Complete blood count and other blood tests were within normal limits, without thrombocytopenia (Table 1). A subsequent cerebral CT and CT-angiography scans did not show any other acute vascular event. Doppler angiography of the carotid artery was performed and showed normal flow without clinically significant plaques, stenoses, occlusions or dissections.

**Table 1:** Blood tests values.

Test	Value	Unit	Reference
Hemoglobin	14.4	g/dl	12.0–16.0
Hematocrit	41	%	36–46
MCHC	36	g/dl	31–37
MCH	30	pg	24–33
MCV	83	fl	80–99
MPV	8.8	fl	7.8–11.0
Erythrocytes	4.87	10^12^/L	4.00–5.20
RDW	12.8	%	11.5–14.5
INR	1.00		0.85–1.15
Platelet Count	187	10^9^/L	140–400
Prothrombin	20	Sec.	21–34
Thrombin time	17	Sec.	14–21
Leucocytes	6.3	10^9^/L	3.6–10.5
Creatinine	1.04	mg/dl	0.50–0.90
GFR	52.1	ml/min/1.73 m^2^	>60.0
LDH	242	u/l	0–289
D-Dimer	0.40	mg/ml	<0.77
Fibrinogen	309	mg/dl	180–400
CRP	4.9	mg/l	0.0–5.0

**MCHC**: Mean corpuscular hemoglobin concentration, **MCH**: Mean corpuscular hemoglobin, **MCV**: Mean corpuscular volume, **MPV**: Mean platelet volume, RDW: Red blood cell distribution width, **INR**: International normalized ratio, **GFR**: Glomerular filtration rate, **LDH**: Lactate Dehydrogenase, **CRP**: C-reactive protein.

Giant cell arteritis could be excluded by normal values of erythrocyte sedimentation rate (ESR 20, 70 mm/hr) and C-reactive protein (4.9 mg/L.). In addition, patient had no headache, no temporal artery tenderness, and no decreased temporal artery pulse.

## 3 Discussion

RAOs are divided into arteritic, due to giant cell arteritis, and non-arteritic, in which thromboembolic events have been reported as potential mechanisms.^3^ There is an overall higher incidence of cardiovascular risk factors in CRAO patients than in the age-matched control population. In some studies, cardiovascular risk factors were found in 78% of RVO patients.^4^ More recently and over the past 12 months, COVID-19 infection has also emerged as a potential risk factor for CRAO due to the hypothesized activation of a prothrombotic vascular endothelial microenvironment and increased hypercoagulability.^5^^,^^6^

In March 2021, 13 cases of sinus or cerebral venous thrombosis in association with the AstraZeneca vaccine were reported in Germany. Some of these cases showed features of heparin-induced thrombocytopenia (HIT)-like syndrome.^7^ Furthermore, increased rates for venous thromboembolism were observed within 28 days of vaccination with AstraZeneca in Denmark and Norway.^2^

Smadja et al. assessed the phenotypes and the time frames of the thromboembolic events following the administration of three different COVID-19 vaccines, and reported that both venous and arterial thrombotic events were observed following various COVID-19 vaccines. In addition, they reported that whilst the time period between vaccination and arterial thrombotic events was comparable in all three vaccines (median 2 days), there was a significant difference in incidence time of the venous thrombotic events between AstraZeneca (median 6 days) and the two mRNA vaccines (median 4 days).^7^ This may correlate with our patient having an arterial thrombotic event two days after vaccination.

## 4 Conclusions

To our knowledge, this is the first case of an isolated CRAO following the administration of the AstraZeneca COVID-19 vaccine. Further studies are needed to evaluate this potential association and identify pathophysiologic relationships between COVID-19 vaccinations and CRAO.

## Patient consent

Consent to publish the case report was not obtained. This report does not contain any personal information that could lead to the identification of the patient.

## Intellectual property

We confirm that we have given due consideration to the protection of intellectual property associated with this work and that there are no impediments to publication, including the timing of publication, with respect to intellectual property. In so doing we confirm that we have followed the regulations of our institutions concerning intellectual property.

## Research ethics

We further confirm that any aspect of the work covered in this manuscript that has involved human patients has been conducted with the ethical approval of all relevant bodies and that such approvals are acknowledged within the manuscript.

## Funding

No funding was received for this work.

## Authorship

All listed authors meet the ICMJE criteria.  We attest that all authors contributed significantly to the creation of this manuscript, each having fulfilled criteria as established by the ICMJE.

We confirm that the manuscript has been read and approved by all named authors.

We confirm that the order of authors listed in the manuscript has been approved by all named authors.

## Contact with the editorial office

This author submitted this manuscript using his/her account in EVISE.

We understand that this Corresponding Author is the sole contact for the Editorial process (including EVISE and direct communications with the office). He/she is responsible for communicating with the other authors about progress, submissions of revisions and final approval of proofs.

We confirm that the email address shown below is accessible by the Corresponding Author, is the address to which Corresponding Author's EVISE account is linked, and has been configured to accept email from the editorial office of American Journal of Ophthalmology Case Reports.

We understand that this author is the sole contact for the Editorial process (including EVISE and direct communications with the office). He/she is responsible for communicating with the other authors, including the Corresponding Author, about progress, submissions of revisions and final approval of proofs.

## Declaration of competing interest

No conflict of interest exists.
